# Detection of *BRAF* V600 Mutations in Melanoma: Evaluation of Concordance between the Cobas® 4800 BRAF V600 Mutation Test and the Methods Used in French National Cancer Institute (INCa) Platforms in a Real-Life Setting

**DOI:** 10.1371/journal.pone.0120232

**Published:** 2015-03-19

**Authors:** Samia Mourah, Marc G. Denis, Fabienne Escande Narducci, Jérôme Solassol, Jean-Louis Merlin, Jean-Christophe Sabourin, Jean-Yves Scoazec, L’Houcine Ouafik, Jean-François Emile, Remy Heller, Claude Souvignet, Loïc Bergougnoux, Jean-Philippe Merlio

**Affiliations:** 1 Department of Pharmacology-Genetics, AP-HP, Saint-Louis Hospital, Paris, France; 2 INSERM UMRS 976 Paris, F-75010, France; 3 Department of Biochemistry, Nantes University Hospital, Nantes, France; 4 Department of Molecular Biology and Biochemistry, Lille Regional University Hospital, Lille, France; 5 Department of Biology—Pathology, Montpellier University Hospital, Montpellier, France; 6 Department of Tumor Biology, Alexis Vautrin Center, Vandoeuvre les Nancy, France; 7 Department of Pathology—Cytopathology, Rouen University Hospital, Rouen, France; 8 Laboratory of Pathology and Cytopathology, Edouard Herriot Hospital, Lyon, France; 9 Department of Biology and Pathology, Gustave Roussy Hospital, Villejuif, France; 10 Department of Cancer Biology, Marseille Nord University Hospital, Marseille, France; 11 Department of Pathology, Ambroise Paré Hospital, Boulogne-Billancourt, France; 12 Department of Microbiology and Molecular Biology, Colmar hospital, Colmar, France; 13 Roche, Boulogne-Billancourt, France; 14 Department of Tumor Biology, Bordeaux University Hospital, Bordeaux, France; Moffitt Cancer Center, UNITED STATES

## Abstract

Vemurafenib is approved for the treatment of metastatic melanoma in patients with *BRAF* V600 mutation. In pivotal clinical trials, BRAF testing has always been done with the approved cobas 4800 BRAF test. In routine practice, several methods are available and are used according to the laboratories usual procedures. A national, multicenter, non-interventional study was conducted with prospective and consecutive collection of tumor samples. A parallel evaluation was performed in routine practice between the cobas 4800 *BRAF* V600 mutation test and home brew methods (HBMs) of 12 national laboratories, labelled and funded by the French National Cancer Institute (INCa). For 420 melanoma samples tested, the cobas method versus HBM showed a high concordance (93.3%; kappa = 0.86) in *BRAF* V600 genotyping with similar mutation rates (34.0% versus 35.7%, respectively). Overall, 97.4% and 98.6% of samples gave valid results using the cobas and HBM, respectively. Of the 185 samples strictly fulfilling the cobas guidelines, the concordance rate was even higher (95.7%; kappa = 0.91; 95%CI [0.85; 0.97]). Out of the 420 samples tested, 28 (6.7%) showed discordance between HBM and cobas. This prospective study shows a high concordance rate between the cobas 4800 *BRAF* V600 test and home brew methods in the routine detection of *BRAF* V600E mutations.

## Introduction

The incidence and mortality rates of melanoma have risen sharply throughout the world over the past few decades and the incidence of melanoma has shown the largest increase of all cancers [1]. Cutaneous melanoma is the most serious skin cancer due to its high potential for metastasis[2,3] and, until recently, no effective treatments were available [4].

Recent discoveries in cell signaling pathways that control cellular proliferation have provided a greater understanding of the biology that underlies melanoma and have elucidated the central role of *BRAF* kinase [5,6]. The mitogen-activated protein kinase (MAPK) pathway is a key regulator of melanoma proliferation and is critical to oncogenic signalling in the majority of patients with cutaneous melanoma. Activating *BRAF* V600 mutations have been shown to occur in 40%–60% of malignant melanomas [7,8], including in recent reports based on analyses of French patients [9,10].

The discovery of such somatic mutations in the *BRAF* gene has paved the way for developing targeted therapies in melanoma [11,12]. Indeed, the importance of targeting this pathway for melanoma treatment using specific *BRAF* inhibitors has been successfully demonstrated in *BRAF* V600-mutated melanoma in preclinical models [13,14] and, more importantly, in clinical trials [15–18].

Vemurafenib (Zelboraf), a selective *BRAF* inhibitor, has been shown to increase the overall median survival by 3.6 months (13.2 months in the vemurafenib arm versus 9.6 months in the dacarbazine arm; HR, 0.37; 95%CI, 0.26 to 0.55) [15] and has recently been approved as a first line therapy in *BRAF* (V600E in the USA, V600 in Europe) mutated advanced melanoma [15,19].

Vemurafenib was granted Marketing Authorization (MA) in Europe in February 2012 for the treatment of adult patients with *BRAF* V600 mutation-positive unresectable or metastatic melanoma. The approval of vemurafenib has made *BRAF* V600 molecular genotyping mandatory requiring molecular diagnostic testing in order to select patients who will benefit from this therapy [15]. Therefore, vemurafenib was developed conjointly with the cobas 4800 *BRAF* V600 Mutation Test (Roche Molecular Diagnostics) using allele-specific real-time polymerase chain reaction (PCR) and TaqMelt technology to determine *BRAF* V600 mutation status in DNA isolated from formalin-fixed, paraffin-embedded (FFPE) tumor tissue [20]. It was designed to detect the predominant *BRAF* V600E mutation with high sensitivity (less than 5% of V600E sequence in a wild-type sequencing environment).

In August 2011, the cobas 4800 *BRAF* V600 Mutation Test reagent obtained European Community-*In Vitro* Diagnostic (EC-IVD) labeling for the detection of the main *BRAF* V600 somatic mutations in routine diagnostic testing. The analytical performances of the reagent have been evaluated in several multicenter studies [21–23].

In France, the French National Cancer Institute (INCa) has set up a national network of 28 regional molecular cancer genetics platforms where selective molecular tests, including *BRAF* V600 genotyping, are routinely performed using methods specific to each laboratory [24,25]. In this real-life study, we evaluated the concordance of the cobas 4800 *BRAF* V600 Mutation Test relative to the home brew methods (HBM) used at 12 participating INCa platforms when tested in parallel for *BRAF* genotyping in melanoma samples.

## Materials and Methods

### Melanoma samples

This national, multicenter, prospective, non-interventional study included 420 consecutive tumor samples of histologically proven melanoma tumor tissue, surgical specimens or biopsies of primary tumors or metastases (regardless of disease stage), fixed and paraffin-embedded. Tumor samples for which the fixative was unknown were excluded and no sample could be included in the study more than once. At selection, 12 INCa platform laboratories equipped with the cobas 4800 System and routinely performing *BRAF V600* mutation testing were invited to participate to the study and eligible tumor samples were consecutively included. The study was conducted in accordance with the recommendations of the professional code of ethics and good epidemiological practices established by the Association of French-speaking Epidemiologists [26]. The solicited Ethics Committee (Comité de Protection des Personnes/CPP) considered that neither patient consent nor CPP approval was required for this non-interventional study. Patients data was anonymized during the conduct of the research in an e-CRF. The BRAF mutation testing was done first using the routine method and secondarily using cobas 4800 automated test for the purpose of the study. The samples dedicated to cobas test were anonymized with a patient code. Data analysis performed by the investigator was completely anonymized. The investigator was not the testing operator. Operators performed the test independently of the investigator.

### *BRAF* V600 Detection Methods

*BRAF* mutation testing was carried out in parallel by the cobas 4800 *BRAF* V600 Mutation Test (Roche Molecular Diagnostics) and the HBMs routinely used by the 12 participating INCa molecular genetics platforms in French hospitals (Boulogne-Billancourt, Colmar, Lille, Lyon, Marseille, Montpellier, Nantes, Paris, Pessac, Rouen, Vandoeuvre les Nancy and Villejuif). The HBMs included direct Sanger sequencing, pyrosequencing [27], allele-specific PCR [10], SNaPshot and high-resolution melting (HRM) analysis followed by Sanger sequencing in cases with abnormal curve [9]. DNA extraction was performed using either manual or automated registered techniques.

**Fig 1:**
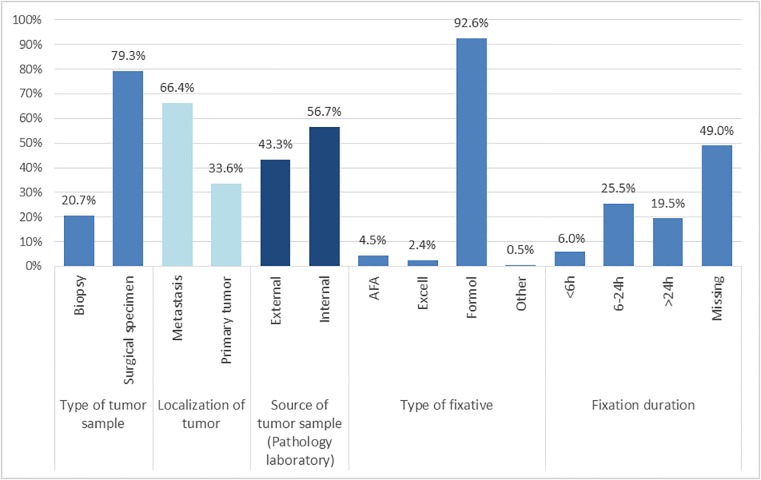
Tumor sample characteristics and method of fixation.

*BRAF* mutation testing with the cobas 4800 *BRAF* V600 Mutation Test was performed by personnel trained in the techniques of PCR and the use of the cobas 4800 System in each center and following the procedures in the Package Insert and in the cobas 4800 System Operator's Manual. The manufacturer’s protocol included another DNA extraction starting from the same tumor sample (mainly FFPE sections), also according to the supplier’s recommendations. The cobas 4800 *BRAF* V600 Mutation Test has been optimized and validated for formalin-fixed paraffin embedded (FFPE) tissues. Other cobas criteria include formaldehyde fixation, slice thickness ≥5 μm, dewaxing, percentage of tumor cells ≥50% and DNA concentration ≥5 ng/μL. However, as the study evaluated the concordance in routine practice, the cobas method was also applied to other types of material with different fixatives (as shown in Fig. 1).

In case of discordance between the local HBM technique and the cobas method, the molecular biologist was responsible for making the decision to either make a cross-over between the two samples of extracted DNA and to reanalyze them by the two different techniques or to perform another analysis (retesting, immunohistochemistry for BRAF V600E, etc) according to his/her usual practices.

### Statistical Analysis

The primary study objective was to assess the concordance between the cobas 4800 BRAF V600 mutation test and the HBMs for detection of *BRAF* V600 mutations in melanoma. The primary endpoint was the discordance rate between the INCa molecular laboratory HBM and the cobas 4800 *BRAF* V600 mutation test for detection of *BRAF* V600 mutations in melanoma. Secondary endpoints were tumor sample characteristics, descriptions of preanalytical and analytical methods, and management of discordant cases. No changes in the analyses were implemented after protocol signature. The scientific committee requested several additional exploratory analyses, including description of DNA in quantitative amounts and qualitative classes, and description of the preanalytical and analytical methods according to the BRAF V600 testing results.

A total of 384 tumor samples would allow to detect a rate of 10% of discordance between the two methods with a precision of 3% (α = 5%) for the 95% confidence interval (CI). The total number of samples to be included in this study was 400, assuming a 5% rate of non-evaluable samples (damaged equipment, too few tumor cells, etc).

The principal analysis was carried out on the population of evaluable samples. Initially, total mutation rates for the routine methods of INCa molecular genetics platform laboratories and for the cobas 4800 *BRAF* V600 Mutation Test were described as "V600 mutation", "No V600 mutation" or "Invalid result" (defined as samples that were evaluable but the results could not be interpreted). A discordance was defined when the result of the usual laboratory method was different from that of the cobas 4800 BRAF V600 Mutation Test. The Kappa coefficient was presented with its 95% CI to evaluate the concordance between the two methods.

A sensitivity analysis was carried out by not taking into account samples giving “invalid results” for at least one of the two methods. Another sensitivity analysis was performed on the subgroup of samples strictly respecting cobas criteria. A two-sided type one error of 0.05 was used for all statistical tests. As planned in the protocol, an intermediate analysis was performed that was not considered in this paper. Due to the descriptive nature of the statistical analysis, the intermediate analysis did not affect the final analysis. No adjustment of the type one error was needed.

## Results

### Melanoma samples

Between December 2012 and April 2013, 420 melanoma samples were consecutively included in this *BRAF* V600 mutation parallel testing study conducted in 12 platform laboratories, with a median number of 32 samples (min 20-max 52) in each laboratory. Most tumor samples were surgical specimens (79%) and 66% were samples of metastases. Tumor specimens were provided by the hospital internal pathology laboratory in 57% of the cases and from other laboratories in the remaining cases (Fig. 1).

### Pre-analysis of tumor samples

The fixation was performed and controlled by the internal pathology laboratory for 56.4% of the samples (Fig. 1). The time from sampling to fixation (ischemia) was less than 2 hours (33.3% of all samples) and duration of fixation was 6–24 hours (25.5% of all samples). The overwhelming majority of samples were formalin-fixed (92.6%) but three centers also used Alcohol-Formol-Acetic acid and two centers also used Excell Plus for fixation.

Most of the samples (90.7%) were subjected to paraffin removal prior to DNA extraction. No necrosis was detected in 74.2% of the samples. The median percentage of tumor cells was 75% and there were ≥ 50% of tumor cells in 91.9% of the samples. Melanin was absent from 51.7% of the samples and was present in moderate to high amounts in 15.9% (Table 1).

**Table 1:** Pre-analytical sample characteristics overall and according to BRAF mutation.

	OVERALLN = 420	BRAF V600 mutationN = 136	No BRAF V600 mutationN = 253	Discordant resultN = 28	Invalid resultsN = 3
**Slice thickness, *N***	*386*	*126*	*230*	*27*	*3*
Median (min-max)	5 (2–20)	5 (3–20)	5 (2–20)	4 (3–10)	5 (4–20)
<5μm	121 (31.3%)	36 (28.6%)	69 (30.0%)	15 (55.6%)	1 (33.3%)
≥5μm	265 (68.7%)	90 (71.4%)	161 (70.0%)	12 (44.4%)	2 (66.7%)
**Percentage of tumor cells**					
Median (min-max)	75 (5–100)	70 (15–100)	80 (5–100)	80 (20–100)	90 (80–90)
<50%	34 (8.1%)	13 (9.6%)	17 (6.7%)	4 (14.3%)	0
≥50%	386 (91.9%)	123 (90.4%)	236 (93.3%)	24 (85.7%)	3 (100%)
**DNA extraction method**					
Automated	188 (44.8%)	57 (41.9%)	110 (43.5%)	19 (67.9%)	2 (66.7%)
Manual	232 (55.2%)	79 (58.1%)	143 (56.5%)	9 (32.1%)	1 (33.3%)
**Percentage of necrosis**, *N*	*341*	*113*	*210*	*16*	*2*
Median (min-max)	0 (0–60)	0 (0–50)	0 (0–60)	0 (0–20)	0 (0–0)
0%	253 (74.2%)	77 (68.1%)	164 (78.1%)	10 (62.5%)	2 (100.0%)
≥0%	88 (25.8%)	36 (31.9%)	46 (21.9%)	6 (37.5%)	0
**Presence of melanin**, *N*	*391*	*7*	*12*	*9*	*1*
Absent	202 (51.7%)	68 (52.7%)	122 (50.6%)	11 (57.9%)	1 (50.0%)
Low	127 (32.5%)	41 (31.8%)	80 (33.2%)	6 (31.6%)	0
Medium	43 (11.0%)	15 (11.6%)	26 (10.8%)	1 (5.3%)	1 (50.0%)
High	19 (4.9%)	5 (3.9%)	13 (5.4%)	1 (5.3%)	0
**DNA elution volume (μL)**					
Median (min-max)	100 (25–185)	100 (25–185)	100 (25–185)	100 (25–185)	60 (50–100)
**DNA concentration (ng/μL),** *N*	*392*	*130*	*233*	*26*	*3*
Median (min-max)	76.5	89.0	75.0	32.3	4.0
	(0.6–1514)	(0.6–1514)	(0.6–1465)	(1.3–468)	(1.5–54)
<5 ng/μL	36 (9.2%)	10 (7.7%)	18 (7.7%)	6 (23.1%)	2 (66.7%)
≥5 ng/μL	356 (90.8%)	120 (92.3%)	215 (92.3%)	20 (76.9%)	1 (33.3%)
**Amount of DNA for HBM (ng) *N***	*392*	*130*	*233*	*26*	*3*
Median (min-max)	6850	7800	6375	3235	240
	(15–102200)	(15–102200)	(15–75500)	(33–37206)	(146–2700)
<5000ng	166 (42.3%)	50 (38.5%)	99 (42.5%)	14 (53.8%)	3 (100%)
≥5000ng	226 (57.7%)	80 (61.5%)	134 (57.5%)	12 (46.2%)	0

DNA extraction was performed manually for 55.2% of the samples using silica-membrane-based nucleic acid purification kits (Table 1). The remaining extractions (n = 188) were performed using automated methods. The instruments used were Qiacube (Qiagen) for 79 samples (42.0%), iPrep (Invitrogen) for 50 (26.6%), Maxwell 16 (Promega) for 30 (16.0%), and Qiasymphony (Qiagen) for 29 (15.4%). Overall, for the HBM, the amount of DNA obtained was at least 5000 ng in 57.7% of the samples and the median amount of DNA was 6850 ng. At least 5000 ng of DNA were obtained in 50.6% of the samples with automated extraction and in 62.5% of the samples with manual extraction. The median amount of DNA was 5175 ng and 8234 ng, for automated and manual extraction methods respectively.

### Description of the analytical methods used in routine practice

The most frequently used HBM *BRAF* tests, each used in three centers, were real-time allele-specific PCR (36.7% of all samples), High Resolution Melting analysis combined with Sanger sequencing (28.1%) and pyrosequencing (16.0%) (Table 2). Overall, the median size of amplicons was 120 bp [70–232 bp] (Table 2).

**Table 2:** Analytical method sample characteristics overall and according to BRAF mutation.

	OVERALLN = 420	BRAF V600 mutationN = 136	No BRAF V600 mutationN = 253	Discordant resultN = 28	IndeterminateN = 3
**HOME BREW ANALYTICAL METHOD**					
**Method of mutation detection**					
Allele-specific/real time PCR	154 (36.7%)	47 (34.5%)	100 (39.5%)	7 (25.0%)	0
HRM + Sanger sequencing	118 (28.1%)	36 (26.5%)	73 (28.9%)	8 (28.6%)	1 (33.3%)
Pyrosequencing	67 (16.0%)	28 (20.6%)	37 (14.6%)	1 (3.6%)	1 (33.3%)
SNaPshot	30 (7.1%)	11 (8.1%)	19 (7.5%)	0	0
Sanger sequencing	51 (12.1%)	14 (10.3%)	24 (9.5%)	12 (42.9%)	1 (33.3%)
**Size of amplicons used (base pairs [bp])**					
Median (min-max)	120 (70–232)	120 (70–232)	120 (70–232)	157 (70–232)	107 (82–221)
**COBAS ANALYTICAL METHOD**					
**Amount of DNA (ng) *N***	*420*	*136*	*253*	*28*	*3*
Median (min-max)	3635	4680	3350	4085	467
	(7–58000)	(7–30000)	(300–58000)	(300–49000)	(450–782)
<5000ng	250 (59.5%)	69 (50.7%)	162 (64.0%)	16 (57.1%)	3 (100%)
≥5000ng	170 (40.5%)	67 (49.3%)	91 (36.0%)	12 (42.9%)	0

A punch was used in four HBM centers (27.9% of all samples) and was used for 10.7% of the samples with the cobas test. For both methods, when there was no punch, one to three sections were used in most cases, with a maximum of 10 sections.

The median amount of DNA obtained with the cobas extraction kit was 3635 ng (min 7 ng—max 58000 ng).

### Concordance between cobas and HBM

The frequency of *BRAF* V600 mutations detected were 34.0% with the cobas vs 35.7% with the HBM methods (Table 3). Wild-type genotypes were observed in 63.3% and 62.9% of the samples using cobas and HBM tests respectively. Overall, the genotyping tests delivered a valid result (either presence or absence of BRAF mutation) in 97.3% of tumors with the cobas and 98.6% of tumors with HBM tests respectively.

**Table 3:** Description of concordances and discordances between the cobas method and home brew methods (all samples N = 420 and samples strictly respecting cobas criteria N = 185).

All samples	Cobas method			
Home brew methods	BRAF V600 mutation	No BRAF V600 mutation	Invalid results	Total
				N = 420
BRAF V600 mutation	136 (32.38%)	**10 (2.38%)**	**4 (0.95%)**	150 (35.7%)
No BRAF V600 mutation	**7 (1.67%)**	253 (60.24%)	**4 (0.95%)**	264 (62.9%)
Invalid results	-	**3 (0.71%)**	3 (0.71%)	6 (1.4%)
Total	143 (34.0%)	266 (63.3%)	11 (2.6%)	
n (%) concordant	392 (93.3%)			
**n (%) discordant**	**28 (6.7%) 95% CI: [4.48%; 9.49%]**			
**Kappa coefficient**	**0.8611 95% CI: [0.8125; 0.9097]**			
**Samples strictly respecting cobas criteria**	**Cobas method**			
**Home brew methods**	BRAF V600 mutation	No BRAF V600 mutation	Invalid results	Total
				N = 185
BRAF V600 mutation	63 (34.05%)	**1 (0.54%)**	**1 (0.54%)**	65 (35.1%)
No BRAF V600 mutation	**3 (1.62%)**	114 (61.62%)	**2 (1.08%)**	119 (64.3%)
Invalid results	-	**1 (0.54%)**	-	1 (0.5%)
Total	66 (35.7%)	116 (62.7%)	3 (1.6%)	
n (%) concordant	177 (95.7%)			
**n (%) discordant**	**8 (4.3%) 95% CI: [1.89%; 8.34%]**			
**Kappa coefficient**	**0.9082 95% CI: [0.8472; 0.9692]**			

A high concordance rate of 93.3% (392 samples) and a kappa coefficient of 0.86 (95% CI [0.81; 0.91]) were obtained on comparing cobas and HBM methods for *BRAF* V600 mutation detection in routine practice (Table 3 —all samples). The concordance and kappa coefficient were even better when taking into account the 389 evaluables samples (concordance rate = 95.8%, kappa = 0.91 and when taking into account the 185 samples strictly respecting the cobas criteria (concordance rate = 95,7%, kappa = 0.91).

With HBM, the main types of mutation detected were V600E in 88.7% of the mutations and V600K in 9.3%, others included V600R (1.3%) and V600D (0.7%). Six samples (1.4%) gave invalid results with the in-house method and 11 (2.6%) with the cobas test.

A further analysis performed taking into account the 185 samples strictly respecting the cobas criteria gave a higher concordance rate of 95.7% and a kappa coefficient of 0.91 (95% CI [0.85; 0.97]) (Table 3 —samples strictly respecting cobas criteria).

### Analysis of the discordant cases

Results from the cobas method and the HBM were discordant for 28/420 samples (6.7%) (Table 3 —all samples). Among these 28 discordant cases, 11 had invalid results (8 samples with the cobas and 3 with HBM) and 17 were true discordant cases (7 with *BRAF* V600 mutation detected by the cobas only and 10 detected by HBM only). For the 10 samples with *BRAF* V600 mutation detected by the HBM but not by the cobas Mutation Test. Five of them were V600K, D or R mutations. Of these 5 cases, all specimens contained ≥50% of tumor cells, except one (40% of tumor cells). Of the other 5 samples with V600E mutation not detected by the cobas Mutation Test, two of the specimens contained only 20% tumor cells. For the 7 other samples with *BRAF* V600 mutation detected by the cobas^,^ but not by HBMs, this result was confirmed after retesting in 4 samples, the other samples were considered as having invalid results (n = 2) or not mutated (n = 1).

Univariate logistic regression analyses performed on tumor samples for which there was discordance between the two methods showed that explanatory factors for discordance may be Sanger sequencing (p<0.0001), slice thickness (>5μm p = 0.0068) and automated DNA extraction (p = 0.014) (Table 4). Multivariate analysis showed that automated DNA extraction method (p = 0.0411) and Sanger sequencing method (p<0.0001) could be two potential explanatory factors of discordance (Table 4).

**Table 4:** Univariate and multivariate analyses of discordant results.

	% Discordant^§^	Univariate analysis		Multivariate analysis	
		OR [95%CI]	*p*-value	OR [95%CI]	*p*-value
**PRE-ANALYTICAL METHOD**					
**Source of tumor sample**					
External pathology lab	5.5%				
Internal pathology lab	7.6%	1.407 [0.633, 3.126]	0.4017		
**Slice thickness (μm) (classes)**					
<5μm	12.4%	2.984 [1.351, 6.589]	0.0068*		
≥5μm	4.5%				
**Percentage of tumor cells (classes)**					
<50%	11.8%	2.011 [0.655, 6.177]	0.2223		
≥50%	6.2%				
**Extraction method**					
Automated	10.1%	2.786 [1.229, 6.311]	0.0141*		
Manual	3.9%			2.487 [1.038;5.964]	0.0411*
**HOME BREW METHODS**					
**Amount of DNA (ng)-pre-analytical**					
<5000ng	8.4%	1.643 [0.739, 3.650]	0.2232		
≥5000ng	5.3%				
**Sanger sequencing**					
No	4.3%			6.264 [2.708;14.487]	<0.0001*
Yes	23.5%	6.788 [2.995, 15.387]	<0.0001*		
**Punch**					
No	6.9%	1.170 [0.484, 2.831]	0.7273		
Yes	6.0%				
**COBAS METHOD**					
**Amount of DNA (ng)-pre-analytical**					
<5000ng	6.4%				
≥5000ng	7.1%	1.111 [0.512, 2.412]	0.7906		
**Punch**					
No	6.9%	1.602 [0.367, 6.985]	0.5307		
Yes	4.4%				

^§^Percentages are calculated on the total number of samples (N = 420);

*The most highly significant values.

Among the 12 centers participating in this study, the discordance rate ranged from 0% to 31%. The highest discordance rates (13.6% and 31.0%) occurred in two centers using Sanger sequencing and long amplicons (157 and 221 bp).

## Discussion

The cobas 4800 *BRAF* V600 Mutation Test has been developed within the strict framework of clinical trials as a companion diagnostic for targeted cancer therapy with vemurafenib, a *BRAF* kinase inhibitor [20].

Here we report on the first prospective and multicenter study assessing in routine practice testing of *BRAF* V600 mutations in melanoma samples by HBM in molecular genetics laboratories in parallel with the cobas test, an approved and automated assay. Results showed a high concordance rate of 93.3% (Kappa = 0.86) between the two assessments.

The rates of *BRAF* V600 mutations observed between the large array of different HBM and cobas tests were very close (35.7% for HBM and 34.0% for cobas). These results are consistent with the INCa platforms annual report for the year 2012 during which 3,479 patients underwent *BRAF* testing and the frequency of mutation detection for the *BRAF* gene was 37.6% [28]. In the INCa report, mutation frequency varied depending on the mutation detection method from 30.6% with TaqMan real-time PCR to 42.7% with allele-specific PCR [28]. Compared to the general situation in INCa platforms, the present study included less laboratories performing direct Sanger sequencing and more tests were performed using allele-specific PCR [29]. Allele-specific PCR, High Resolution Melting analysis combined with Sanger sequencing, or pyrosequencing were used in the centers with the highest annual activity for malignant melanoma testing.

Overall, the rates of invalid results were low, at 1.4% and 2.6% for the HBM and cobas tests respectively, compared to the 5.1% reported in the INCa report [28]. The INCa report showed a variation in the frequency of invalid results according to the mutation detection method from 2.5% with allele-specific PCR to 7.0% with direct Sanger sequencing.

Among the 10 cases of discordant *BRAF* V600E genotypes, it is difficult to conclude whether this discordance is due to possible contaminations, tumor heterogeneity or to other factors such as DNA quality or preanalytical parameters. The characteristics of the preanalytical and analytical methods were mostly similar for samples whatever the *BRAF* V600 mutation result (V600 mutation, no V600 mutation, discordant or invalid result). Indeed, any attempts to highlight specific preanalytical parameters to explain the discordance are hindered as ischemia and fixation time parameters were missing for about half of the samples. Also, potential contaminations during automated DNA extraction, although unlikely, cannot be excluded. Intra-tumoral heterogeneity should also be taken into account. Indeed, using laser microdissection followed by mutation specific Snapshot assay, a substantial proportion of individual tumor specimens were found to contain a mixture of BRAF mutant and wild-type melanoma cells [30]. In addition, a low percentage of tumor cells may possibly explain one case of discordance according to the molecular technique sensitivity threshold.

As expected, based on preclinical and clinical studies involving retrospective sequencing, rare V600R, V600D and V600K mutations were detected with low sensitivity by cobas [22,31,32]. In a study of 295 melanoma samples comparing the cobas 4800 BRAF V600 Mutation Test with direct Sanger sequencing assays, the cobas test was less sensitive for mutations other than the single-nucleotide V600E mutation [33]. In this retrospective analysis of BRAF V600 mutations, twenty samples were excluded because of invalid results (by cobas (n = 3), sequencing (n = 15), or both (n = 2)) and 23 samples with positive sequencing results gave negative cobas results [33].

Tumors with other V600 mutations, such as V600K, are sensitive to vemurafenib treatment [34]. In our study, only 2 discordant samples had V600K mutations, as detected by HBM but not with cobas.

Concerning the analytical genotyping method used, a higher discordance rate was observed in centers using direct Sanger sequencing with long amplicons for mutation detection. Low sensitivity (15–30%) of the Sanger sequencing method has previously been reported [22,23,35,36]. Furthermore, false positive detections by the Sanger method could be due to contamination or background noise [35]. Conversely, high concordance was observed between the cobas method and the High Resolution Melting analysis technique followed by Sanger sequencing.

Interestingly, both pyrosequencing and High Resolution Melting analysis methods have been shown by two French groups participating in this study to provide concordant data with immunohistochemical detection of the BRAF p.V600E protein when applying robust and simple interpretation criteria [9,27]. Although rare false negative cases exist using immunohistochemistry as a screening tool, these cases would be tested with DNA-based methods in a hierarchical algorithm [37]. On the other hand, "false positive" cases detected by immunohistochemistry were reported wild type using direct sequencing, but further detected as mutated using Pyrosequencing [37]. Altogether our molecular comparison between cobas and HB methods also indicated that direct sequencing should not be used in routine testing of melanoma samples as well as in technical comparison since this generates a high rate of discordant cases.

In conclusion, the results of this first prospective study comparing the cobas 4800 BRAF V600 Mutation Test and various HBM developed in national platforms in France allows us to draw 2 main conclusions: standard DNA sequencing (especially using long DNA amplicons) generates a significant proportion of invalid results, and the cobas 4800 BRAF V600 mutation assay is efficient for testing V600E BRAF mutations.
